# Supplementary material for: WordCommentsAnalyzer: A windows software tool for qualitative research
Source: F1000Res. 2018 Sep 4;7:536. Originally published 2018 May 3. [Version 2] doi: 10.12688/f1000research.14819.2 (PMC6137411; doi:10.12688/f1000research.14819.2)
Supplement: Supplementary file 3 [file f1000research-7-17614-s0002.tgz › b2a79496-4ada-47c4-9682-a99ce867836d.zip!/Eight Iranian Health Journals/The Journal of Toloo-e-Behdasht_8.docx]

© The Journal of Toloo-e-Behdasht. This work is licensed under CC BY 4.0 (https://creativecommons.org/licenses/by/4.0/).

Volume & Issue: دوره 15 - شماره 6

Title: اسپور کلستریدیوم بوتولینوم و آلودگی قارچی در عسل های تولیدی ایران

http://tbj.ssu.ac.ir/article-1-888-fa.html

مقدمه: عسل ماده غذایی بسیار غنی و انرژی بخش حاصل از شهد گیاهان توسط زنبور عسل است. در عسل احتمال وجود میکروارگانیسم ها به شکل اسپور وجود دارد. یکی از این میکروارگانیسم های خطرساز باکتری کلستریدیوم بوتولینوم است که توانایی بروز بوتولیسم به ویژه در نوزادان را دارد. این مطالعه به منظور بررسی میزان شیوع آلودگی عسل های تولیدی مناطق مختلف ایران به اسپور کلستریدیوم بوتولینوم و کپک و مخمر صورت گرفت. روش بررسی: این مطالعۀ توصیفی مقطعی در سال 1392 بر روی تعداد 130 نمونه عسل، تهیه شده از زنبورستان های کشور انجام گرفت. فراوانی اسپور کلستریدیوم بوتولینوم در عسل های ایران با استفاده از شوک حرارتی و محیط کشت گوشت پخته شده گرمخانه گذاری به مدت 10 روز در دمای 35 درجه سانتی گراد مورد بررسی قرار گرفت. باکتری کلستریدیوم پرفرنژنس PTCC:1766 به عنوان نمونه ی کنترل مثبت استفاده شد. آزمون شمارش کپک و مخمر نیز طبق استاندارد ملی ایران  انجام پذیرفت. یافته ها: نتایج نشان داد اسپور کلستریدیوم بوتولینوم در هیچ یک از  نمونه های مورد مطالعه حضور نداشت. آلودگی به کپک و مخمر در 34 نمونه (67/26%) بالاتر از حد مجاز استاندارد بود. استان کردستان با 6/66% آلودگی بالاتر از حد مجاز، بیشترین آلودگی و مازندران با دامنه آلودگیCFU/g  55-33 کمترین  میزان آلودگی به کپک و مخمر دارا بودند. نتیجه گیری: عسل های ایران در مقطع زمانی مورد مطالعه از نظر اسپور کلستریدیوم بوتولینوم ایمن بوده اما آلودگی به کپک و مخمر در عسل های مناطق غرب کشور بیشتر و شمال کشور کمترین میزان را دارا می باشد.

Title: سنجش اثر بخشی مداخله آموزشی بر پیشگیری از بیماریهای قلبی-عروقی در بین نوجوانان پسر : کاربرد مدل اعتقاد بهداشتی

http://tbj.ssu.ac.ir/article-1-2127-fa.html

مقدمه: بیماری های قلبی – عروقی از علل اصلی مرگ و میر در جهان می باشند. با توجه به تغییر شیوه زندگی، بسیاری از عوامل خطر بیماریهای قلبی– عروقی از دورانهای کودکی و نوجوانی آغاز می شود، بنابراین این مطالعه با هدف سنجش اثر بخشی مداخله آموزشی مبتنی برمدل اعتقاد بهداشتی بر پیشگیری از بیماریهای قلبی- عروقی طراحی و اجرا گردید. روش بررسی: این پژوهش به صورت یک مطالعه نیمه تجربی از نوع قبل و بعد می باشد که بر روی 180نفراز دانش آموزان پسر  دوره اول متوسطه شهرساری در سال1393 انجام گرفت که به صورت تخصیص تصادفی به دو گروه90  نفره مداخله  و مقایسه تقسیم شدند. سپس قبل از مداخله و 6 ماه بعد ار مداخله مورد ارزیابی قرار گرفتند. ابزار گردآوری اطلاعات پرسشنامه دو بخشی اطلاعات دموگرافیک وابزار سنجش پیشگیری از بیماری های قلبی عروقی بر اساس سازه های مدل اعتقاد بهداشتی بود. تجزیه و تحلیل داده ها با استفاده از نرم افزار   SPSS نسخه 20 و توسط آزمون تکرار مشاهدات، تی تست مستقل،کای اسکوئر انجام گرفت. در این مطالعه سطح معنی داری آزمون 05/0 در نظر گرفته شد. یافته ها: نتایج حاصل از مداخله آموزشی نشان داد که قبل از اجرای مداخله آموزشی اختلاف آماری معنی داری بین دو گروه مداخله و مقایسه از نظرسازه های مدل اعتقاد بهداشتی وجود نداشت(005/0p>). در گروه مداخله اختلاف میانگین نمرات آگاهی درک شده(001/0p<)، حساسیت درک شده(001/0p<)، شدت درک شده(001/0p<)،منافع (001/0p<) و موانع درک شده (002/0p=)، خودکار آمدی (001/0p<) قبل و 6 بعد از مداخله از نظر آماری معنی دار بود به گونه ای که بعد از اجرای مداخله میانگین نمرات سازه های مدل اعتقاد  بهداشتی افزایش یافته بود. نتیجه گیری: به نظر می رسد این مدل می تواند موانع ادراکی را تا حدودی از بین ببرد، بعلاوه  حساسیت ، شدت و منافع موضوع را پر رنگ نماید و نهایتا خودکارآمدی دانش آموزان را درزمینه پیشگیری از بیماری قلبی عروقی ارتقاء دهد.

Title: بررسی تأثیر عوامل محیطی و وجود پوسیدگی زودرس دوران کودکی روی شمارش استرپتوکوک موتانس بزاق کودکان 3-5 ساله شهر یزد

http://tbj.ssu.ac.ir/article-1-1754-fa.html

مقدمه: پوسیدگی زودرس دوران کودکی  یکی از شایعترین مشکلات کودکان است که در 3-45درصد جمعیت پیش دبستانی جهان مشاهده میشود.عوامل میکروبیال یکی از چهار جز اصلی (زمان، میکرو ارگانیسم، رژیم غذایی و میزبان)برای ایجاد پوسیدگی هستند.استرپتوکوک های موتانس از مهم ترین میکرو ارگانیسم های پوسیدگی زا هستند هدف از این مطالعه  تعیین میزان کلنی های استرپتوکوک موتانس در بزاق کودکان با پوسیدگی زودرس دوران کودکی و مقایسه آن با میزان کلنی های استرپتوکوک موتانس در بزاق کودکان بدون پوسیدگی و همچنین بررسی اثر عوامل محیطی روی تعداد این کلنی ها می باشد. روش بررسی: در این مطالعه میزان کلنی استرپتوکوک موتانس 76 کودک 3-5 ساله در دو گروه با پوسیدگی زودرس دوران کودکی و بدون پوسیدگی بررسی شده است. جمع آوری بزاق  به روش غیر تحریکی و کشت آن روی محیط کشت اختصاصی استرپتوکوک موتانس انجام شد.پس از شمارش کلنی ها داده ها با استفاده از تست های آماری Chi-Square, و spearman و  Kruskal– Wallis, Mann– Whitney تجزیه و تحلیل شد. یافته ها:اختلاف تعداد کلنی در گروه های مورد مطالعه از نظر آماری معنی دار و در کودکان بدون پوسیدگی کمتر از گروه دیگر بود (001/0p<). تفاوت تعداد کلنی ها در گروه های مورد مطالعه بر حسب سن، جنس،وزن، نمایه توده بدنی (BMI)، تحصیلات پدر و مادر تفاوت معنی داری نشان نمیدهد ولی تعداد دفعات مصرف مواد قندی، روش تغذیه (شیشه شیر یا شیر مادر) شیر دهی شبانه باعث اختلاف معنی دار در تعداد کلنی ها شد(019/0p = ). نتیجه گیری: در مطالعه حاضر تفاوت معنی داری از لحاظ میزان کلنی های استرپتوکوک موتانس بین دو گروه مشاهده شد.رابطه تعداد دفعات مصرف مواد قندی و روش تغذیه با میزان کلنی ها معنی دار بود.میکرو ارگانیسم هاهنوز مهم ترین عامل در بروز پوسیدگی هستند ولی الگوی تغذیه و دفعات مصرف مواد قندی هم نقش بسزایی ایفا میکند. همچنین نتایج این مطالعه نشان میدهد میزان تحصیلات والدین نقشی در کاهش پوسیدگی نداشته که باید به این موضوع توجه ویژه ای داشت.

Title: دیدگاه خبرگان مالیاتی و محیط زیستی شهر یزد در خصوص تأثیر برقراری نظام مالیات سبز بر کاهش آلودگی های زیست محیطی

http://tbj.ssu.ac.ir/article-1-2304-fa.html

مقدمه:استفاده صحیح و کارا از منابع مختلف انرژی و منابع طبیعی لازمه دستیابی به رشد اقتصادی در راستای توسعه پایدار است. بر این اساس اهمیت حفظ و حراست از منابع تجدید ناپذیر و کاهش اثرات منفی فعالیت های صنعتی و اقتصادی بیش از پیش برای دولت ها مشهود می باشد. بدین منظور مطالعه ای تحت عنوان " بررسی دیدگاه خبرگان مالیاتی و محیط زیستی شهر یزد در خصوص برقراری نظام مالیات سبز وکاهش آلودگی های زیست محیطی"  صورت پذیرفت. روش بررسی: در این مطالعه توصیفی برای گردآوری اطلاعات، پرسشنامه ای با مقیاس پنج گزینه ای لیکرت طراحی و توسط 126 نفر از خبرگان مالیاتی و محیط زیستی که به روش نمونه گیری تصادفی طبقه ای انتخاب شده بودند، تکمیل گردید. اطلاعات استخراج شده توسط نرم افزار SPSS و با استفاده از آزمون t - student مورد سنجش و ارزیابی قرار گرفت. یافته ها:با توجه به اینکه هر سه آماره بدست آمده از آزمونt  برای آلودگی هوا، آب و خاک از مقدار بحرانی جدول در سطح آلفای 5 درصد (645/1= 05/0z ) بزرگتر می باشد، طبق نظر کارشناسان با اطمینان 95% می توان بیان کرد که برقراری نظام مالیات سبز می تواند موجب کاهش هر سه نوع آلودگی زیست محیطی هوا، آب و خاک گردد. نتیجه گیری: بر اساس نتایج، استقرار نظام مالیات سبز می تواند از طریق کاهش انواع آلودگی های زیست محیطی در ارتقاء سطح سلامت، رفاه اجتماعی و وضعیت اقتصادی جامعه نقش بسزایی داشته باشد. لازم بذکر است عوامل افزایش مالیات بر ارزش افزوده بنزین، مواد شوینده، سموم و آفت کش ها بر خلاف سایر عوامل نمی تواند کاهش آلودگی زیست محیطی را به همراه داشته باشد.

Title: ارزیابی کیفیت خدمات غیر بالینی بیمارستانی با استفاده از تکنیک مبتنی بر طبقه بندی: مطالعه مقطعی در یکی از بیمارستان های تهران

http://tbj.ssu.ac.ir/article-1-1957-fa.html

مقدمه:در فرآیند اندازه‌گیری کیفیت، نیاز به یک ابزار موثر در شناسایی و اولویت بندی معیارهای مربوط به ارزیابی کیفیت خدمات، محسوس است. تعیین جذابیت با استفاده از تکنیک مبتنی بر طبقه بندی (مک بث) که یکی از روش‌های تصمیم گیری چند معیاره می‌باشد؛ می تواند در امر پیچیده سنجش کیفیت خدمات، پژوهشگران را یاری نماید. روش‌بررسی:مطالعه حاضر یک پژوهش مقطعی است که شامل سه مرحله اصلی می باشد. مرحله اول استخراج شاخص‌های سنجش کیفیت با استفاده از مطالعه مروری جامع واخذ نظرات خبرگان نظام سلامت، مرحله دوم وزن دهی به شاخص‌ها و مرحله نهایی رتبه بندی بخش‌های بستری یکی از بیمارستان‌های دانشگاه علوم پزشکی تهران با استفاده از تکنیک مک بث و با نمونه ای 300 نفری از بیماران بستری بود. یافته‌ها:هشت حیطه اصلی (پاسخگویی، ایمنی و مدیریت بحران، ملموسات، توجه به بیمار، ارتباطات سلامت، شایستگی و حرفه ای بودن، دسترسی، اعتبار و تضمین) برای سنجش کیفیت گزینش شد که شایستگی دارای بیشترین و دسترسی دارای کمترین وزن بودند. همچنین بخش داخلی دارای بالاترین و بخش اورولوژی دارای کمترین کیفیت خدمات غیر بالینی بودند. نتیجه گیری:استفاده از تکنیک های ارزیابی مناسب که تفاوت در کیفیت خدمات بخش‌ها و بیمارستان‌های مختلف را ارزیابی و با یکدیگر مقایسه کرده و ضمن تعیین نقاط قوت و ضعف این بخش‌ها و بیمارستان‌ها در ابعاد مختلف به آن‌ها در بهبود خدمات خود یاری دهد، یکی از نیازهای اساسی واحد‌های ارزیابی کیفیت مستقر در بیمارستان‌ها، دانشگاه‌ها و وزارت بهداشت می‌باشد.

Title: بررسی رابطه بین الگوهای ذهنی کارکنان و توانمندسازی آنها در دانشگاه علوم پزشکی شهر یزد

http://tbj.ssu.ac.ir/article-1-1488-fa.html

مقدمه: توانمندسازی به علت توجه کافی به مراتب نیاز کارکنان، به منظور دستیابی به رشد بیشتر و در نتیجه تأثیر آن  بر بهره وری و کارایی سازمان، مورد توجه سازمان ها قرار گرفته است. بنابراین هدف این پژوهش تبیین جایگاه و نقش الگو های ذهنی و تعیین رابطه آن با توانمندسازی کارکنان دانشگاه علوم پزشکی شهید صدوقی یزد است. روش بررسی: روش تحقیق، توصیفی از نوع همبستگی است، به منظور جمع آوری داده ها از پرسشنامه ای شامل 51گویه، 36 گویه مربوط به الگو های ذهنی برگرفته از پرسشنامه دانایی فرد وهمکاران(3) با ابعادهدف گرایی،وحدت گرایی،برنامه گرایی، ثبات گرایی، تعادل گرایی وعملکرد گرایی و 15 گویه مربوط به توانمند سازی کارکنان به وسیله پرسشنامه اسپریتزر و میشرابا ابعاد ویژگیهای شایستگی، خودمختاری، تاثیرگذاری، معنی دار بودن و اعتماد، استفاده شده است. پایایی و روایی پرسشنامه ها مورد سنجش قرار گرفت و تایید گردید. تجزیه و تحلیل داده های گردآوری شده با نرم افزار 19SPSS  انجام پذیرفت. یافته ها: یافته های این پژوهش نشان داد که بین الگوهای های ذهنی کارکنان و توانمند سازی آنها در دانشگاه علوم پزشکی شهید صدوقی یزد رابطه معنا داری وجود دارد. همچنین بین مؤلفه های هدف گرایی، وحدت گرایی، برنامه گرایی، تعادل گرایی و توانمندسازی کارکنان دانشگاه علوم پزشکی یزد رابطه معنی داری وجود دارد، ولی بین دو مؤلفه ثبات گرایی و عملکرد گرایی کارکنان وتوانمندسازی آنها رابطه معنی داری وجود ندارد. نتیجه گیری: با توجه به موضوع تحقیق و وجود رابطه معنادار و مثبت بین چهار مؤلفه الگوهای ذهنی کارکنان و توانمندسازی آنها در دانشگاه علوم پزشکی، مدیران سازمان می توانند با تقویت الگوهای ذهنی کارکنان، باعث بهبود توانمندسازی کارکنان شوند.

Title: مقایسه سوگیری حافظه رویدادی بیماران اسکیزوفرن، وابسته های زیست شناختی و افراد عادی

http://tbj.ssu.ac.ir/article-1-1274-fa.html

مقدمه: آنچه احساس مداومت و پیوستگی در ما ایجاد میکند حافظه است و دیدگاه خاصی که هرکس درباره خود دارد به همین حافظه بستگی دارد. در بین دیدگاهها مختلف روانشناسی، فقط روانشناسی شناختی به بررسی و شناخت حافظه پرداخته است. بنظر می رسد حالتهای روحی و روانی برروی حافظه اثر دارد و سوگیری حافظه به سمتهای خاص را منجر میشود. یافته های پژوهشی، در بیماران اسکیزوفرن تحریف های فضایی وجود دارد که این مطلب با نشانه های اصلی بیماران اسکیزوفرن یعنی اختلال در افکار این بیماران همسو است و سوگیری نقش اساسی در ایجاد و پردازش اطلاعات هماهنگ با افکار در بیماران اسکیزو فرن و افراد عادی دارد. هدف پژوهش حاضر بررسی اینگونه سوگیری های حافظه در جهت گزینی به محرکهای خوشایند، ناخوشایند و خنثی می باشد. روش بررسی: اطلاعات استفاده شده در مطالعه حاضر مربوط به واکنشهای بیماران اسکیزوفرن، وابسته های زیست  شناختی و افراد عادی به ماده های خوشایند، ناخوشایند و خنثی آزمون ویلیامز میباشد. این داده ها پس از جمع آوری، ابتدا میانگین، واریانس و انحراف استاندارد آن محاسبه گردید و سپس بوسیله ازمون تحلیل واریانس (ANOVA)  مورد تجزیه و تحلیل قرار گرفت.  یافته ها: در واکنش به محرکهای خوشایند، ناخوشایند و خنثی ازمون ویلیامز بین دو میانگین بیماران وافراد عادی نسبت به محرکهای خوشایند تفاوت معنی داری وجود دارد. یعنی افراد عادی محرکهای خوشایند رابیشتر ازبیماران اسکیزوفرنی جهت گزینی نمودند.همچنین افراد عادی محرکهای خنثی را با اندکی تفاوت بیشتر از بیماران اسکیزوفرن انتخاب کردند. تنهادر محرکهای ناخوشایند بین افراد عادی و بیماران اسکیزوفرن تفاوت معنی داری وجود ندارد. نتیجه گیری: توانایی وانسجام فکری افراد منجر به عملکرد خوب فکری میشود و منجر به این می شود تاحتی افراد همه خاطرات من الجمله خاطرات ناخوشایند رابا جزییات بیشتری بیاد اورند بر همین اساس بنظرمی رسد جهت گزینی حافظه، به سمت حالات روانشناختی حاکم بر فرد می باشد.

Title: اثر بخشی آموزش معنا گرایی اسلامی بر مسئولیت پذیری دانشجویان دانشکده پیراپزشکی دانشگاه علوم پزشکی شهید صدوقی یزد

http://tbj.ssu.ac.ir/article-1-2458-fa.html

مقدمه: با توجه به مطالعات محدود در زمینه مداخلات آموزشی و تاثیر آن بر مسئولیت پذیری، این مطالعه با هدف تعیین اثربخشی آموزش معناگرایی اسلامی بر مسئولیت پذیری دانشجویان دانشکده پیراپزشکی دانشگاه علوم پزشکی شهید صدوقی یزد طراحی و اجرا گردید. روش بررسی: پژوهش از نوع مداخله ای با طرح پیش آزمون- پس آزمون با یک گروه کنترل انجام گرفت. با استفاده ازروش نمونه گیری تصادفی خوشه­ای چند مرحله­ای 30 نفر از دانشجویان دانشکده پیراپزشکی دانشگاه علوم پزشکی شهید صدوقی یزد در سال 95-94 وارد تحقیق شده و بصورت تصادفی به دو گروه آزمون (15 نفر) و کنترل (15 نفر) تقسیم شدند. ابزار گردآوری اطلاعات، پرسشنامه مسئولیت پذیری دانشجویان بود که پیش از اجرای آموزش به عنوان پیش آزمون برای هر دو گروه آزمایش و کنترل اجرا شد. دوره آموزشی معنا گرایی اسلامی بر اساس قرآن وتفاسیر مربوطه تهیه گردیده و  به مدت 8 جلسه دو ساعته برای گروه آزمایش اجرا گردید. در نهایت پس آزمون برای هر دو گروه انجام و داده ها با استفاده از روش­های آمار استنباطی، آزمون تحلیل کواریانس چند متغیره تحلیل شد.  یافته ها: قبل از مداخله بین میانگین نمرات مسئولیت پذیری گروه آزمایش و کنترل تفاوت معنی داری مشاهده نشد ولی بعد از انجام مداخله تفاوت معنی داری دیده شد. به عبارت دیگر آموزش معناگرایی اسلامی موجب افزایش مسئولیت پذیری در دانشجویان دانشکده پیراپزشکی دانشگاه علوم پزشکی شهید صدوقی یزد گردیده است( اثر همپراش 002/ = p، گروه 005/ = p). نتیجه گیری: با توجه به اینکه در این مطالعه، آموزش معناگرایی اسلامی منجر به ارتقاء مسئولیت پذیری دانشجویان گردید لذا بنظر می رسد این نوع آموزش می تواند یکی از راههای ارتقای عزت نفس و بالطبع افزایش مسئولیت پذیری در قشر دانشجو باشد.

Title: بررسی تاثیر سلامت سازمانی بر تعلق خاطر کاری پرستاران در بیمارستان تامین اجتماعی شهر یزد

http://tbj.ssu.ac.ir/article-1-2407-fa.html

مقدمه: فقدان تعلق خاطر کاری در بین پرستاران اثرات سوئی روی بهداشت و سلامت جامعه دارد. بنابراین شناسایی عوامل تاثیرگذار بر این متغیر و تلاش در جهت بهبود آن ضرورت می‌یابد. یکی از عوامل موثر بر تعلق خاطر کاری می تواند سلامت سازمانی بیمارستان باشد. در این راستا هدف پژوهش حاضر بررسی تاثیر سلامت سازمانی بر تعلق خاطر کاری پرستاران است. روش بررسی: تحقیق حاضر از لحاظ هدف کاربردی و از لحاظ روش، توصیفی از نوع پیمایشی و از نظر زمانی مقطعی است که در شش ماهه اول سال 1394 انجام شد. جامعه آماری این تحقیق کلیه پرستاران بیمارستان تامین اجتماعی شهر یزد می باشد. روش نمونه گیری، نمونه گیری تصادفی ساده است. حجم نمونه با استفاده از فرمول کوکران 152 تعیین شد. برای سنجش متغیر ها و بررسی رابطه آنها از دو پرسش نامه سلامت سازمانی نامه لایدن و کینگل (2000) و پرسش نامه تعلق خاطر کاری شافلی و همکاران (2002) استفاده شد. برای سنجش روایی پرسش نامه ها از روش روایی محتوا و تحلیل عاملی تاییدی و برای سنجش پایایی، از روش آلفای کرونباخ و پایایی مرکب استفاده گردید. اطلاعات بدست آمده از پرسش نامه ها از طریق نرم افزارهای SPSS و Smart PLS مورد بررسی و تجزیه و تحلیل قرار گرفته است. یافته ها: نتایج مدلسازی معادلات ساختاری نشان داد که سلامت سازمانی و همه ابعاد یازده گانه آن بر تعلق خاط کاری پرستاران تاثیر مثبت و معنا دار دارد. از میان یازده بعد سلامت سازمانی، سه بعد ارتباطات، انسجام هدف و مشارکت سلامت سازمانی بیشترین اثر مستقیم را بر تعلق خاطر کاری پرستاران دارد. نتیجه گیری: با ارتقاء شاخص های سلامت سازمانی در بیمارستان، می توان تعلق خاطر کاری پرستاران را افزایش داد. با توجه به بالا بودن تاثیر سه بعد ارتباطات، انسجام هدف و مشارکت بر تعلق  خاطر  کاری  پرستاران،  پیشنهاد   می شود مدیران مجموعه با سرمایه گذاری روی این سه بعد، تعلق خاطر کاری پرستاران را در بیمارستان افزایش دهند.

Title: بررسی و مقایسه برخی شاخص های عملکردی بیمارستان قبل و بعد از اجرای بسته درمانی طرح تحول نظام سلامت در بیمارستانهای منتخب دانشگاه علوم پزشکی بوشهر

http://tbj.ssu.ac.ir/article-1-1748-fa.html

مقدمه: در اغلب کشورها به ویژه کشورهای درحال توسعه، بیمارستان به عنوان یک سازمان بسیار هزینه بر محسوب می شود . طرح تحول نظام سلامت را با سه رویکرد حفاظت مالی از مردم، ایجاد عدالت در دسترسی به خدمات سلامت و ارتقای کیفیت خدمات در بیمارستان ها از اردیبهشت ماه سال 93 اجرا شد. در این پژوهش تلاش شد برخی شاخص های عملکردی بیمارستان های منتخب استان بوشهر قبل و بعد از اجرای طرح مورد ارزیابی و مقایسه قرار گیرد. روش بررسی: این پژوهش از مطالعات توصیفی- مقطعی است. ابزار جمع آوری داده ها، شاخص های عملکردی بیمارستان است. جمعیت مورد مطالعه 4 بیمارستان بالای 100 تخت وابسته به دانشگاه علوم پزشکی بوشهر است. جمع آوری و تحلیل داده ها با استفاده از نرم افزار  EXCEL 2013 است. شاخص ها مورد تایید و روایی و پایایی آن قبلا تایید شده است. یافته ها: یافته ها حاکی از آن است که طرح تحول سلامت بر شاخص درصد اشغال تخت و متوسط اقامت روند افزایشی داشته است، شاخص نسبت گردش تخت و وقفه در گردش تخت نیز با تغییرات ناچیزی روبه بهبود بوده اند. نتیجه گیری: با توجه به اینکه شاخص های مورد بررسی در اغلب بیمارستان ها، بعد از اجرای طرح تحول نظام سلامت وضعیت بهتری نسبت به قبل از آن داشته اند و نیز با توجه به اینکه طرح تحول نظام سلامت به تازگی اجرا شده و درمرحله ارزیابی و اصلاح می باشد لذا به نظر می رسد تداوم، ارزشیابی و اصلاح مستمر این طرح می تواند به بهبود شاخص های عملکردی بیمارستان ها بیا نجامد.

Title: اثر پرتو دهی بر ویژگی های عملکردی صمغ کتیرا

http://tbj.ssu.ac.ir/article-1-885-fa.html

مقدمه: پرتودهی، یک تیمار فیزیکی است که طی آن به منظور بهبود ایمنی و کیفیت، محصول را در معرض پرتوهای یونیزان نظیر پرتوهای گاما و ایکس قرار می دهند. هیدروکلوئیدها، ترکیباتی هستند که به منظور بهبود ویژگی‌های بافتی در صنایع غذایی اهمیت زیادی دارند. عملکرد هیدروکلوئیدها به ویژگی هایی ساختاری این ترکیبات بستگی دارد. قرار گیری در معرض پرتو، ممکن است ویژگی‌های ساختاری و عملکردی ترکیبات در معرض را دچار تغییراتی کند. با توجه به اهمیت پرتودهی برای سالم سازی هیدروکلوئیدها در کاربردهای غذایی هدف از این مطالعه بررسی اثر پرتودهی در دوز‌های مختلف بر ویژگی‌های عملکردی صمغ کتیرا در کاربرد‌های غذایی است. روش بررسی: اثر پرتودهی بر ویژگی‌های رئولوژیکی، پارامترهای توصیف کننده اندازه ذرات، پتانسیل زتا و کشش سطحی پراکنش5/0% وزنی- وزنی صمغ کتیرای پرتو دیده در دوز‌های مختلف ( صفر، 75/0، 3، 5 کیلوگری) بررسی شد. به منظور بررسی اثر پرتودهی بر ویژگی‌های رئولوژیکی از رئومتر استفاده شد. اثر پرتودهی بر توزیع اندازه ذرات، پتانسیل زتا و کشش سطحی پراکنش به ترتیب با استفاده از پارتیکل سایزر، زتا سایزر و تنسیومتر تعیین شد. کلیه آزمون‌ها در سه بار تکرار انجام شد. جهت بررسی تأثیر هر یک از فاکتورها از آزمون آنالیز واریانس یک طرفه استفاده شد. مقایسه میانگین‌ها با استفاده از آزمون چند دامنه ای دانکن در سطح احتمال 5 درصد انجام گرفت. یافته‌ها: پرتودهی، ویژگی‌های رئولوژیکی و اندازه ذرات پراکنش محتوی صمغ کتیرا را تغییر می‌دهد، پتانسیل زتا تحت تأثیر پرتودهی در میزان دوز 75/0 کیلوگری افزایش اما ادامه پرتودهی در دوز‌های بالاتر منجر به کاهش آن می‌شود. کشش سطحی پراکنش تحت تأثیر پرتودهی تغییر نمی کند. نتایج حاصل از پارامترهای مورد مطالعه نشان داد، پرتودهی با تغییر ساختار بر عملکرد آن اثر می‌گذارد. میزان این تغییرات به دوز پرتودهی بستگی دارد. نتیجه‌گیری: پرتودهی صمغ کتیرا، با تأثیر بر ساختار در مواردی ممکن است منجر به بهبود عملکرد شود.

Title: هیپوتیروئیدی مادرزادی و عوامل مرتبط بر آن در نوزادان متولد شده 94-93 در استان یزد

http://tbj.ssu.ac.ir/article-1-2385-fa.html

مقدمه : بیماری کم کاری تیروئید نوزادان از علل مهم و قابل پیشگیری عقب ماندگی ذهنی محسوب می شود و با انجام غربالگری نوزادان در اولین روزهای زندگی شانس تشخیص زودرس بیماری و پیشگیری از کاهش ضریب هوشی فراهم می گردد. این مطالعه با هدف تعیین شیوع هیپوتیروئیدی و برخی عوامل موثر برآن انجام گردید تا راهنمایی برای برنامه ریزان و مدیران عرصه بهداشت و درمان باشد. روش بررسی: این مطالعه توصیفی–تحلیلی، بر روی 51938  نوزاد زنده متولد شده در استان یزد از فروردین ماه 1393 تا پایان سال 1394 به صورت سرشماری انجام گردید. TSH نمونه خون پاشنه پای نوزادان بر روی کاغذ  در آزمایشگاه مرجع استان به روش الیزا اندازه گیری شد . برای نوزادان 7-3 روزه  و 8 روزه و بیشتر به ترتیب میزان TSH  کمتر از 5 و 4 میلی مول بر لیتر در گروه سالم و سایر نتایج در گروه مشکوک قرار  گرفته و تا تشخیص نهایی پیگیری شدند. سن، جنس، ملیت، تاریخ و وزن هنگام تولد نوزاد و نیز سن، نوع زایمان مادر و نسبت فامیلی والدین با پرسشنامه جمع آوری گردید. برای اهداف توصیفی از میانگین، انحراف معیار، فراوانی و درصد و برای اهداف تحلیلی از آزمون کای اسکوئر با استفاده از نرم افزار  Epi Info استفاده گردید. سطح معنی داری در تمام موارد کمتر از 05/0 در نظر گرفته شد. یافته ها: از 51938 نوزاد متولد استان یزد 1/51 % پسران بوده و 9/95%  نوزادان بررسی شده ایرانی و بقیه غیر ایرانی بود ه اند .  5/93 % متولدین در شهرها ساکن بوده و 1/26 % والدین با یکدیگر نسبت فامیلی دارند . 7/6 درصد نوزادان در هنگام تولد وزن زیر 2500 گرم داشتند . مادران 29-20 سال با  4/58  % بیشترین گروه مادران را به خود اختصاص داده و  7/45%  نوزادان با سزارین به دنیا آمده اند.  شیوع هیپوتیروئیدی نوزادی برابر با 4/3 در هزار تولد زنده  بوده است که فراوانی ابتلا با وزن هنگام تولد ؛ روش زایمان و شهرستان محل سکونت  ارتباط معنی دار آماری داشته و با فصل تولد، سن مادر، ملیت  و جنس ارتباط نداشته است . نتیجه گیری: شیوع بالای ابتلا ضرورت توجه بیشترین محققین و مدیران نظام سلامت برای تعیین علل ابتلا بیماری در نوزادان به تفکیک نوع گذرا و دایمی و نیز بررسی کیفیت اجرای برنامه غربالگری و بازنگری احتمالی در فرایند اجرا را می طلبد.
